# Anxiolytic Effect of* Citrus aurantium* L. in Crack Users

**DOI:** 10.1155/2017/7217619

**Published:** 2017-10-18

**Authors:** Gabriel Chaves Neto, João Euclides Fernandes Braga, Mateus Feitosa Alves, Liana Clébia de Morais Pordeus, Sócrates Golzio dos Santos, Marcus Tullius Scotti, Reinaldo N. Almeida, Margareth de Fátima Formiga Melo Diniz

**Affiliations:** ^1^Postgraduate Program in Neuroscience Cognitive and Behavior, Federal University of Paraíba, João Pessoa, PB, Brazil; ^2^Department of Nursing and Collective Health, Federal University of Paraíba, João Pessoa, PB, Brazil; ^3^Postgraduate Program in Natural and Synthetic Bioactive Products, Federal University of Paraíba, João Pessoa, PB, Brazil; ^4^Institute of Research in Drugs and Medicines, Federal University of Paraíba, João Pessoa, PB, Brazil

## Abstract

The objective of this study was to investigate the anxiolytic effects of the essential oil (EO) of* Citrus aurantium *L. in patients experiencing crack withdrawal. This was developed with internal users in therapeutic communities in Paraíba, Brazil. The test population consisted of 51 volunteers, subdivided into three groups. To elicit anxiety, the Simulated Public Speaking (SPS) method was used. Physiological measures were assessed at specific phases during the experiment using appropriate equipment. Psychological measures of anxiety were assessed using the Trait-State Anxiety Inventory (IDATE) and the Analog Smoke Scale (HAS). EO was administered by nebulization. The experiment was developed in individual sessions and consolidated to four phases. The results demonstrated that the test subjects in the groups that were given the EO maintained controlled anxiety levels during SPS, when compared to the Control Group (no treatment). Subjects who used the EO also maintained levels of “discomfort” and “cognitive impairment” during SPS. It was concluded that individuals who are experiencing internal crack cocaine withdrawal present high anxiety traits and that nebulization of the EO of* Citrus aurantium *L. provided an acute anxiolytic effect in crack cocaine users exposed to SPS.

## 1. Introduction

Chemical dependency is a widely discussed phenomenon, since abusive use of psychoactive substances has become a serious social and public health concern. Throughout the twentieth century, this problem has gained increased relevance in the world and today is characterized as a chronic disease classified among psychiatric disorders [1].

*Crack *is one of the distinct forms of cocaine, a substance extracted from the leaf of a plant called coca (*Erythroxylum coca*), which is found in the Andes. When the drug is smoked in crack form, a large amount of cocaine molecules reach the brain almost immediately following use, producing an explosive effect. This speed of action is due to the fact that the smoke begins in the lungs, which are highly vascularized organs, and this quickly transports the drug to the brain. The drug is, subsequently, rapidly eliminated from the body, producing a sudden interruption of the sense of wellbeing, followed immediately by immense displeasure and an overwhelming desire to reuse the drug [2].

The use of* crack *has increased due to the high potential for addiction, pleasant effects, easy administration, low cost, and not being injected (becoming a safer route to HIV infection), among other reasons. This high dependence potentially triggers the craving (or cracking) effect. In the specific case of crack dependence, craving is an uncontrollable phenomenon by users, leading them to compulsive use with a daily pattern of consumption, which continues for several days in a row. This is only concluded when the physical, psychological, or financial exhaustion is achieved [3].

*Crack *addicts seeking treatment for drug withdrawal encounter a battle during abstinence, a period that is surrounded by anxiety and an intense craving for drug use. If there is not adequate monitoring, as well as therapeutic management of these manifestations, users eventually return to the use of crack [4].

Anxiety often presents itself as a state of tension, apprehension, and discomfort, involving emotional and physiological factors [5]. Anxiety disorders have been related both to hyperactivity in the amygdala and to a decreased hippocampal response. The pharmacological treatment of anxiety consists of conventional drugs, such as benzodiazepines and antidepressants. Additionally, barbiturates, carbamates, noradrenergics, antihistamines, glutamic acid, and buspirone are other commonly used therapies. Although a number of medications are presented, treatment is still plagued with limitations, side effects, and dependence and often does not have a standardized success rate across the population [6].

Aromatherapy, which consists of the therapeutic application of essential oils (EO) by inhalation, has been quite effective in relieving anxiety symptoms in research conducted in Brazil [7, 8]. In the United States, the use of herbal and medicinal plants by the population varies from 16.5 to 42.0%, with 5.5 to 20.5% being used for anxiety-related conditions [9]. Traditionally, populations of several countries use preparations based on Citrus species in the treatment of nervous system disorders, especially anxiety or insomnia [10].

Among the species,* Citrus aurantium *L. is popularly known as “bitter orange” [11], and its EO is rich in limonene [12]. Data has indicated that using* Citrus aurantium *L. EO has achieved results indicating anxiolytic effects, both in animals and human studies [11, 13, 14]. These findings highlight a new path in the scientific field for novel research evaluating results that corroborate and amplify the knowledge about the EO of* Citrus aurantium *L. and its anxiolytic effects in diverse population groups.

There is a high number of crack dependents in the world, and they face numerous difficulties to remain in treatment. Therefore, it is necessary to search for alternative measures that aid and contribute to the effectiveness of treatment. The objective of the present study was to investigate the anxiolytic potential of* Citrus aurantium *L. EO as a complementary therapy to reduce anxiety in patients enduring the withdrawal of crack cocaine.

## 2. Materials and Methods

This is an experimental study of an acute pharmacological clinical trial, controlled and randomized, and was developed at the Federal University of Paraíba (UFPB), Postgraduate Program in Neuroscience Cognitive and Behavior [16]. The population groups consisted of nonusers of crack cocaine. This study occurred in two therapeutic communities of recovery and treatment of chemical dependents in the state of Paraíba, Brazil, with voluntary users of* crack *in abstinence.

The study was approved under protocol number 094/1115. CAAE: 42619715.2.0000.5188 from the Ethics and Research Committee of the Health Sciences Center of the Federal University of Paraíba, in compliance with Resolution number 466/12 of the National Health Council, which regulates the conduct of research involving human beings.

### 2.1. Experimental Substance

The substance used for inhalation was the essential oil (EO) of* Citrus aurantium* L., produced and marketed by the company “By Samia Aromatherapy.” The species* Citrus aurantium* L., popularly known as bitter orange, laranjeira‐amarga, or laranjeira cavalo, is a native plant of Southeast Asia, introduced in Brazil in the period of colonization [17].

The EO was administered by nebulization, 2 drops (0.1 mL) of EO in 1.9 mL of distilled water solution with an emulsifier (Tween 80 at 12%), for each subject. The subjects of the “Control Group” experienced the same procedure; however, they received only the distilled water with an emulsifier. An electric nebulizer inhaler (Inalar®) was used; each group had an inhalation kit, exempting remnants of the EO during administration in the “Control Group.” Following each experiment, the inhalation kits underwent a disinfecting process [18].

### 2.2. Gas Chromatography

In order to confirm the composition of the* Citrus aurantium* L. EO and guarantee product quality, an analysis was performed at the Pharmaceutical Products Quality Control Laboratory at the UFPB. Chromatograms were obtained by gas chromatography (Shimadzu GC-MS-QP5050A) using a 5% phenyl and 95% dimethylpolysiloxane capillary column with a length of 30 m, 0.25 mm internal diameter, and 0.25 *μ*m film thickness, manufactured by J & W Scientific (Santa Clara, CA, USA).

### 2.3. Participants

The population consisted of 51 volunteers, who were subdivided into three groups according to the treatment. The “Control Group,” non-crack users who were not internal to the therapeutic communities (*n* = 17), and two experimental groups, the “Nonuser EO Group,” non-crack users who were not internal to the therapeutic communities (*n* = 17), and the “User EO Group”, who were users of crack that were internal to the therapeutic communities (*n* = 17).

The eligibility criteria of the “User EO Group” included: presenting with a chemical dependence, being internally abstinent and having crack as a drug reason for hospitalization, male gender, being older than 18 years old, not making use of substances that affect the central nervous system, lack of cardiovascular problems, lack of upper airway obstruction problems, and lack of neurological and/or psychiatric comorbidities that affect cognition. The subjects of the “Control Group” and “Nonuser EO Group” were randomly selected in the general population; the subjects have a sociodemographic profile similar to the “EO User Group” group, following the same eligibility criteria; however, they are subjects without chemical dependence and are not internal to the therapeutic community.

### 2.4. Experimental Anxiety Induction Model

To induce anxiety, the Simulated Public Speaking (SPS) method [19] was used. The SPS has been shown to cause physiological and psychological changes. Briefly, the subject is requested to deliver a speech in front of a video camera with its image being displayed on a TV screen. The speech, with a fixed time of 4 minutes, should describe anxious moments in your life.

### 2.5. Psychological Measures of Anxiety Assessment

To assess anxiety levels, the State Trait Anxiety Inventory (IDATE), an inventory developed by Spielbergert et al. (1970) [20], was translated and validated for the Portuguese language by Biaggio and Natalício (1979) [21]. It is a tool composed of two self-assessment subscales: the IDATE-Trait (IDATE-T), which defines the trait of anxiety of the individual and differentiates the tendency to react to situations identified as threatening. This is intended to be a more stable characteristic. The second subscale is the state IDATE (IDATE-E), which identifies the state of anxiety in relation to a situation considered anxious or distressing and is intended to be a transitory characteristic. Each of the subscales presents 20 questions, with four possible degrees of intensity of response, ranging from 1 to 4, in which the scores added by each volunteer oscillate between 20 and 80 points.

To accurately measure levels of anxiety, cognitive impairment, sedation, and discomfort, the Humor Analog Scale (HAS), a self-assessment scale originally proposed by Norris (1971) [22], was translated and validated for the Portuguese language By Zuardi and Karniol (1981) [23]. Consisting of 16 items, each composed of a straight line of 100 mm connecting two adjectives of opposite directions; the center of the line corresponds to the habitual state of the individual.

### 2.6. Physiological Measures for Anxiety Assessment

Physiological measures were measured during specific phases of the experiment using appropriate equipment. The model I-330-C2 + Plus Clinical System (J & J Engineering®) was used for the measurements of End Temperature (TEMP) and levels of electric conductance of the skin (ECS). The measurements of systolic blood pressure (SBP), Diastolic Blood Pressure (DBP), and heart rate (HR) were measured using the pulse sphygmomanometer (TeshLine®).

### 2.7. Procedures

The experiment was developed for individual sessions. The locations of the therapeutic communities provided adequate rooms to perform the experiment on volunteer users of crack. The “Nonuser EO Group” and the “Control Group” had the sessions developed in a room provided by the Health Sciences Center, UFPB. Prior to initiating the experiment, brief explanations were provided discussing the objectives of the study and free and informed consent of the participants was obtained; the subjects were not informed about the type of OE to be inhaled and the purpose of it. A semistructured interview was conducted to characterize the sample population and to identify variables such as, age, sex, drug consumption, and time of consumption. The clinical trial was consolidated to four phases: (I) Basal, (II) Stressor, (III) During, and (IV) Final, adapted from the model of Guimarães et al. (1987) [19]. The four phases are described below.

*Baseline Phase (BP)*. The IDATE-T, IDATE-E, and HAS were measured along with the physiological measures of SBP, DBP, HR, TEMP, and ECS. The differentiation between the groups occurred at the end of this phase. The “Nonuser Group” and the “User EO Group” participants inhaled* Citrus aurantium L*. EO by nebulization for 5 minutes while the “Control Group” subjects inhaled only the distilled water with emulsifier by nebulization during the same time period. 

*Stressor Phase (SP)*. The subjects were informed they would have two minutes to prepare a speech focusing on situations that contributed to anxiety during their lives and four minutes to deliver the speech in front of a video camera having their image displayed on a TV. Following the two minutes of preparation and prior to the beginning of the speech, the IDATE-E, the HAS, and the physiological parameters (SBP, DBP, HR, TEMP, and ECS) were measured. 

*During Phase (DP)*. Following the two minutes of speech, the subject was interrupted and the IDATE-E and HAS measured the physiological parameters (SBP, DBP, HR, TEMP, and ECS), and, quickly after collecting the data, the speech was resumed. 

*Final Phase (FP)*. The IDATE-, HAS, and the physiological parameters (SBP, DBP, HR, TEMP, and ECS) were measured fifteen minutes following the end of the discourse.

### 2.8. Statistical Analysis

Statistical analysis was performed with the help of the Graph Pad Prism statistical software (version 6.00, Graph Pad Software Inc., San Diego, CA, USA). Hypothesis tests were defined according to the normality of the data and the classification of variables, using parametric methods (ANOVA, followed by the Bonferroni test) and nonparametric methods (Kruskal-Wallis, followed by Dunn's).

Data were presented with mean and standard error of the mean (e.o.m.) for the parametric methods and in the median and percentiles (25–75th percentile) for the nonparametric, when *P* < 0.05 was considered significant.

## 3. Results

### 3.1. Analytical Control of Essential Oil of* Citrus aurantium *L

**Figure 1:**
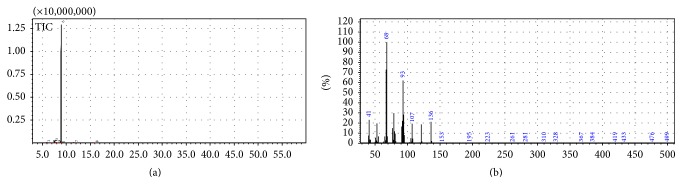
(a) Chromatogram of the essential oil of* Citrus aurantium* L. (By Samia); (b) limonene mass spectrum, showing the base peak at 68* m/z*.

Figure 1(a) illustrates the peaks of the analyzed compounds. Limonene displayed the highest peak, indicating its role as the major compound. Data demonstrated a retention time of 8.9 min and area 48124161, corresponding to 97.99% of the essential oil analyzed.  Figure 1(b) shows the mass spectrum of the limonene compound having a molecular weight of 136* m*-*z*, and a base peak of 68* m*-*z*. The Kovats index (1029) was calculated and compared with the literature according to Table 1.

### 3.2. Characterization of Individuals Participating in the Study

The “Control Group” had a mean age of 28 years (±2.01), the “Nonuser EO Group” had a mean age of 24 years (±0.7282), and the “User EO Group” had a mean age of 30 years (±2,125). Anxiety-Trace scores resulted in the “User EO Group” presenting with the highest score, a median of 45 (37–57), followed by the “Control Group” with a median of 41 (38–53), and “Nonuser EO Group” with 37 (30–40).

### 3.3. Evaluation of the Effects of the Essential Oil of* Citrus aurantium *L. and Placebo on the Psychological Parameters Measured

When analyzing the anxiety levels measured by the IDATE-E and the HAS, similar variations of the scores are observed, which reinforces the confidence of the measurements. The IDATE-E scores between the phases of Simulated Public Speaking are presented in Table 2; the groups presented uniform levels of anxiety in the baseline phase (BP), with no significant differences observed. In the Stressor Phase (SP), a significant difference was observed between the groups, where the groups treated with the EO had controlled anxiety levels, while the “Control Group” demonstrated an increase in these levels. In the During Phase (DP), only the “Nonuser EO” group maintained a significant difference against the “Control Group.” At the Final Phase (FP), the groups had similar medians, with no significant differences.

The Cognitive Impairment Factor scores did not indicate a significant difference between the groups in the BP. In the EP, the groups treated with the EO presented a significant difference in comparison to the “Control Group” (*P* = 0.0018). In the DP, only the “Nonuser EO Group” maintained a statistical difference (*P* = 0.0075) when compared to the “Control Group”; the “User EO Group” did not display a statistical difference in relation to the “Control Group.” In the FP, no statistical difference between groups was observed (Table 3).

The discomfort factor presented a score with no statistical difference between the groups in the BP (Table 3). In the EP and DP, a statistical difference was identified between the “Nonuser EO Groups” and “Control Group.” However, the “User EO Group” did not present statistical differences in comparison to the “Control Group” at any time. Regarding the sedation factor, no variations were observed that could be attributed to the inhalation of the EO.

### 3.4. Evaluation of the Effects of the Essential Oil of* Citrus aurantium *L. and Placebo on the Measured Physiological Parameters

Regarding the physiological parameters evaluated, Table 4 presents the variation of the SBP. The groups did not display significant differences in the BP. In the EP, the “Control Group” experienced an increase in mean SBP and a significant difference was observed between the “Control Group” and the “Nonuser EO Group.” Although the “User EO Group” did not display a statistical difference in relation to the “Control Group” in the EP, the mean of the SBP was reduced. In the DF group, a statistical difference between the “Control Group” and the “Nonuser EO Group” was observed. In the FP, all groups presented with a lower mean SBP, although no statistical differences were found.

The physiological measures of Diastolic Blood Pressure, Heart Rate, Electrical Conductance of the Skin, and End Temperature were also measured and analyzed. However, there were no significant differences between the variables in any of the test groups at any of the phases evaluated that could be attributed to the inhalation of the EO.

## 4. Discussion

Anxiety, specifically generalized anxiety disorder (GAD), has increased in drug users [24]. Crack users are also presenting with increased anxiety disorders at a high frequency. These disorders are identified in studies as a frequent comorbidity among users and regular use of the substance presents a significant relationship with the presence of anxiety disorders [25–27].

It has been previously reported that crack users demonstrate increased anxiety levels. Studies indicate that younger individuals have higher anxiety scores compared to older ones [28, 29]. In the present study, results indicated that a mean age of 30 years presented with greater anxiety levels than what had previously been reported. The data indicated that active crack users had the highest average of trait anxiety scores when compared to the “Nonuser EO Group” and the “Control Group.”

Crack results in an increase in dopamine concentration in the reward system at a much greater level than natural stimuli. The repeated activation of the reward system generates a learning mechanism that modulates behavior in a progressive way to seek the drug. In a short period of time, which varies between individuals and amount of consumption, the individual enters into a neurophysiological exhaustion of sensations of reward and sensitization of the mesolimbic pathways. This subsequently compromises additional neurobiological systems; among the systems, we can mention that the hypothalamic-pituitary-adrenal system is normally activated during drug withdrawal. Alteration of this system is directly related to changes in the state of anxiety and stress and, depending on the intensity and frequency, alters the anxiety traits of the individual [30].

Crack users also try to control the drug-obsessed quest and anxiety with the help of individual harm reduction strategies. In the studies conducted, users have reported strategies for craving relief and pharmacological and behavioral tactics to avoid their development such as, eating, having sex, playing soccer, working, avoiding the social context of using crack, and using drugs that cause drowsiness [31].

During the experiment, the groups that received* Citrus aurantium *L. EO by inhalation, “User EO Group” and “Nonuser EO Group,” demonstrated lower scores of the IDATE-E during the EP of the SPS. Conversely, the “Control Group” showed a higher score and a significant difference with the other groups during that phase. Although the crack user group is more likely to react anxiously to a situation identified as threatening, anxiety is maintained at controlled levels at the time of SPS intervention.

Levels of anxiety measured by HAS in the “Control Group” were increased during the EP and MP, with a significant difference in comparison to the groups that inhaled the EO. The “Nonuser EO Group” had reduced anxiety levels in the EP and these levels remained controlled in the DP. The “User EO Group” presented a slight increase in anxiety levels in the EP and DE. The difference in anxiety levels of the groups reinforces the anxiolytic hypothesis of inhaled* Citrus aurantium *L. EO prior to the SPS experiment. Although the participants in this study did not know which EO they were inhaling, they stated that the EO odor was not unknown to them. As pleasant aromas may induce a state of wellbeing in people, do not rule out a possible placebo effect.

It is reasonable to believe that the anxiolytic effects observed in this study were due to EO; nonclinical studies have shown anxiolytic effects of inhaled* Citrus aurantium *L. EO in rats. The effect was observed on the behavior of the animals when developing specific tests [13, 14]. In one study, in addition to observing significant anxiolytic activity of the EO, they presented results that strongly suggest the involvement of 5-HT1A receptors, a subtype of serotonin receptors, presenting a possible pathway of action [10].

A clinical study performed on patients awaiting dental care consisted of exposing subjects to the inhalation of orange EO in the waiting room. Subjects who were exposed to the EO were found to be calmer with a lower level of anxiety state [32]. Results indicated anxiolytic properties of sweet orange EO in healthy subjects while developing an anxiogenic task, evidenced by the significant difference in state anxiety levels between the group exposed to the aroma and the Control Group [8].

Clinical trials that used* Citrus aurantium *L. on anxiety reduction obtained satisfactory results [33, 34]. Preoperative patients received a* Citrus aurantium *L. bloom two hours prior to the procedure, using the IDATE as an instrument for measuring anxiety and, comparing with the Control Group, the authors identified a reduction in preoperative anxiety in outpatient surgery of the experimental group [33]. Additionally, a study performed on patients with chronic myeloid leukaemia exposed patients to the EO prior to the procedure for collecting medullary material. Data indicated that patients who inhaled the EO* Citrus aurantium *L. presented a decrease in the IDATE-E score and remained relaxed during the procedure. EO, even used in only a single dose, presented similar performance levels to the anxiolytic diazepam and demonstrated efficacy in the anxiety control of patients undergoing an unpleasant diagnostic procedure [11].

This study focused on crack users; the EO also presented an anxiolytic effect in a group that lives daily with anxiety at different levels, one of the largest problems experienced in the period of abstinence and maintenance of drug abandonment. Research has been performed to evaluate alternative anxiety control methods that could be utilized by crack users. Cooperative games and respiratory relaxation have been effective in reducing cravings and anxiety levels in addicted crack users, and the results allow a novel therapeutic approach, suggesting viable and effective strategies for the management of cravings and anxiety symptoms in crack dependents [3, 34, 35].

Cognitive impairment was another factor measured by HAS. The Control Group presented increased cognitive impairment at test times, which was different from the EO treated groups. The “User EO Group” maintained a level of cognitive impairment at the Stressor Phase and decreased at the During Phase. The “Nonuser EO Group” reduced the level at the two intermediate stages of the test.

The increase in anxiety levels reflects the cognitive changes that impair the individual's performance against certain tasks. The results demonstrate that individuals who inhaled the EO of* Citrus aurantium *L. showed no change in cognitive impairment. The effect of EO on crack users is highlighted, since drug abuse causes cognitive alterations. Results identified neurocognitive impairments in crack dependents, such as changes in attention tests, verbal fluency, visual memory, verbal memory, learning ability, and executive functions [36].

Anxiety is accompanied by a sense of discomfort due to the anticipation of danger or something that is unknown. During SPS, the “Control Group” indicated an increase in discomfort levels with a significant difference in comparison to the “Nonuser EO Group.” Throughout the phases of the test, although the “User EO Group” did not present with statistical differences compared to the “Control Group,” the crack users began at a higher level of discomfort than the other groups and reduced this level in the following phases. The EO of* Citrus aurantium *L. controlled the levels of anxiety, in addition to allowing the individuals to remain comfortable during the accomplishment of the anxiogenic task.

The physiological measures evaluated indicated that SPS produced experimental anxiety, as demonstrated in previous studies [6, 37], evidenced by changes from the baseline values of each measurement, mainly between the BP and EP. A transient emotional state, such as anxiety, is strongly marked by tension, apprehension, and activation of the autonomic nervous system and tends to increase blood pressure, heart rate, skin conductance, and lower extremity temperature [38, 39].

Among the measured physiological measures, only SBP presented variations that could be attributed to the inhalation of* Citrus aurantium *EO. In one study, a decrease in SBP was observed by the group exposed to* Citrus aurantium *L. EO, with a similar effect to the group that received diazepam [11]. Based on the results presented, researchers observed satisfactory results of* Citrus aurantium *L. EO in decreasing the parameters of DBP and HR, postulating that the effects of EO on the physiological parameters suggest a decrease in autonomic excitability. Aromatherapy appears to modulate the activities of the autonomic nervous system toward equilibrium [40].

## 5. Conclusion

By analyzing the results obtained in this clinical trial, it can be concluded that individuals who experience crack withdrawal present a high anxiety trait. Administration by nebulization of* Citrus aurantium *L. EO is indicated to be efficient in controlling the psychological parameters of anxiety in individuals exposed to an anxiogenic task. As for the physiological measures, only the SBP of the “Nonuser EO Group” remained constant during the test. The EO of* Citrus aurantium *L. showed no effect on the additional measured physiological measures, suggesting that the pathways of the EO action do not have an effect on the autonomic system.

The EO of* Citrus aurantium *L., administered by nebulization, showed an acute anxiolytic effect in crack users in abstinence. This previously unreported finding has great clinical relevance when presenting a viable alternative of complementary therapy in the control of anxiety in users who are abandoning the use of drugs. Additional studies are required to increase the knowledge of the use of aromatherapy, the duration, and the time of action of the antianxiety effect of the EO in the control of anxiety in users of crack at times other than withdrawal.

## Figures and Tables

**Table 1:** Identification of the major compound [15].

Name	Peak	Retention time	Area	Area%	Kovats index, calculated	Kovats index, literature
Limonene	68	8,9 min	48124161	97,99	1029	1031

**Table 2:** Presentation of the Medians (Percentile 75%, Percentile 25%) of the IDATE-E scores between the phases of the Simulated Public Speaking (SPS).

Groups	Phases of application of SPS, median (Percentile)			
	Basal	Stressor	During	Final
Anxiety				
Control	38 (29–43)	46 (36–58)	38 (35–52)	33 (30–35)
OE Nonuser	33 (30–37)	32^1^ (29–37)	34^2^ (30–36)	32 (29–35)
OE User	36 (32–40)	34^1^ (31–39)	38 (31–40)	31 (30–35)
*P* value	0,1732	0,0023	0,0279	0,9555

*Source: Direct Research 2015*.* Statistical Test:* Kruskal-Wallis and Dunn's posttest. ^1^Significantly different from the “Control Group” at the time Stressor. ^2^Significantly different from the “Control Group” at the time.

**Table 3:** Presentation of the mean and standard error (Ep) of the HAS factors (anxiety, cognitive impairment, and discomfort) in the different phases of the Simulated Public Speaking (SPS).

Groups	Phases of application of the SPS, mean (Ep)			
	Basal	Stressor	During	Final
*Anxiety*				
Control	33 (4,921)	50 (5,615)	44 (6,058)	26 (4,28)
OE Nonuser	26 (4,213)	23^*∗*^ (3,369)	27^#^ (4,834)	23 (4,389)
OE User	29 (3,829)	35^*∗*^ (3,52)	34 (3,014)	25 (3,409)
*P* value	0,5603	0,0002	0,0530	0,8730
*Cognitive impairment*				
Control	37 (2,123)	46 (5,314)	41 (5,152)	35 (4,147)
OE Nonuser	30 (3,928)	25^*∗*^ (2,873)	22^2^ (3,293)	23 (3,71)
OE User	30 (3,212)	30^*∗*^ (3,66)	29 (3,709)	30 (3,579)
*P* value	0,1893	0,0018	0,0075	0,0937
*Discomfort*				
Control	25 (2,925)	35 (3,665)	35 (3,755)	25 (3,254)
OE Nonuser	21 (2,296)	21^1^ (2,425)	22^2^ (2,558)	20 (2,244)
OE User	31 (4,767)	28 (4,571)	29 (4,574)	27 (4,937)
*P* value	0,1746	0,0344	0,0361	0,3051
*Sedation*				
Control	35 (3,306)	26 (3,056)	29 (3,393)	26 (3,851)
OE Nonuser	32 (3,661)	30 (4,811)	25 (4,076)	25 (3,946)
OE User	34 (4,837)	37 (5,715)	37 (5,094)	34 (5,521)
*P* value	0,4426	0,3428	0,1201	0,3066

*Source: *Direct Research 2015. *Statistical Test:* ANOVA and Bonferroni posttest. ^1^Significantly different from the “Control Group” at the time Stressor. ^2^Significantly different from the “Control Group” at the time. *∗* is equivalent to number 1. # is equivalent to number 2.

**Table 4:** Presentation of the mean and standard error (Ep) of the systolic blood pressure in the different phases of the Simulated Public Speaking (SPS).

Groups	Phases of application of SPS, mean (Ep)			
	Basal	Stressor	During	Final
*SBP*				
Control	130 (3,146)	141 (3,997)	140 (3,127)	130 (3,285)
OE Nonuser	125 (3,122)	125^1^ (2,516)	125^2^ (3,113)	121 (2,361)
OE User	135 (2,942)	133 (3,338)	135 (2,806)	130 (3,149)
*P* value	0,0744	0,0071	0,0041	0,0624

*Source*: Direct Research 2015. *Statistical Test:* ANOVA and Bonferroni posttest. ^1^Significantly different from the “Control Group” at the time Stressor. ^2^Significantly different from the “Control Group” at the time.
